# Flame Retardancy of Carbon Fibre Reinforced Sorbitol Based Bioepoxy Composites with Phosphorus-Containing Additives

**DOI:** 10.3390/ma10050467

**Published:** 2017-04-27

**Authors:** Andrea Toldy, Péter Niedermann, Ákos Pomázi, György Marosi, Beáta Szolnoki

**Affiliations:** 1 Department of Polymer Engineering, Faculty of Mechanical Engineering, Budapest University of Technology and Economics, Budapest H-1111, Hungary; atoldy@mail.bme.hu (A.T.); niedermann@pt.bme.hu (P.N.); pomazia@pt.bme.hu (Á.P.); 2 Department of Organic Chemistry and Technology, Faculty of Chemical Technology and Biotechnology, Budapest University of Technology and Economics, Budapest H-1111, Hungary; bszolnoki@mail.bme.hu

**Keywords:** carbon fibre reinforced bioepoxy composite, phosphorus-containing additive flame retardant, solid- and gas-phase mechanism, synergism

## Abstract

Carbon fibre reinforced flame-retarded bioepoxy composites were prepared from commercially available sorbitol polyglycidyl ether (SPE) cured with cycloaliphatic amine hardener. Samples containing 1, 2, and 3% phosphorus (P) were prepared using additive type flame retardants (FRs) resorcinol bis(diphenyl phosphate) (RDP), ammonium polyphosphate (APP), and their combinations. The fire performance of the composites was investigated by limiting oxygen index (LOI), UL-94 tests, and mass loss calorimetry. The effect of FRs on the glass transition temperature, and storage modulus was evaluated by dynamic mechanical analysis (DMA), while the mechanical performance was investigated by tensile, bending, and interlaminar shear measurements, as well as by Charpy impact test. In formulations containing both FRs, the presence of RDP, acting mainly in gas phase, ensured balanced gas and solid-phase mechanism leading to best overall fire performance. APP advantageously compensated the plasticizing (storage modulus and glass transition temperature decreasing) effect of RDP in combined formulations; furthermore, it led to increased tensile strength and Charpy impact energy.

## 1. Introduction

The need for the development of polymers originating from renewable resources is increasing with the decreasing amount of the mineral oil stock and with the spreading of environmental awareness. This tendency has emerged also in the polymer composite industry, including such demanding sectors as automotive and aircraft applications [1], where high performance thermosetting materials (such as epoxy resins) are applied. Besides the environmental advantages, the partial or full replacement of polymer matrices and reinforcements by renewable ones can reduce the dependence on petrochemicals and eliminate the effect of their fluctuating price level. The use of natural fibres as reinforcement in bio-based matrices seems to be an obvious solution [2]. However, several issues need to be addressed when applying natural fibres, i.e., high moisture-absorption [3], strict conditioning requirement before processing to avoid the formation of micro-voids [4], limited processing temperatures due to low thermal stability, and fluctuating properties depending on the year and place of the harvest, extraction method, etc. [5]. These disadvantages hinder their spread in high-tech applications such as the aircraft industry, where the use of stiff parts is indispensable. Accordingly, in these demanding sectors, synthetic reinforcements of constant properties, mostly carbon fibres, are applied. Although the research interest on renewable matrix materials has exponentially increased in the last few years [6,7], and some bio-based epoxy resins offer an alternative to benchmark diglycidyl ether of bisphenol A (DGEBA) in terms of mechanical properties [5], until now only a few publications dealt with the performance of bioepoxy composites reinforced with carbon fibres [8,9,10,11].

Compared to metallic structures, the main drawback of the polymer composites, including the ones prepared from renewable resources, is their highly flammable organic matrix. In order to fulfil the strict safety requirements of high-tech applications, effective flame retardation of the composites has to be elaborated while maintaining mechanical properties at an appropriate level [12,13]. Phosphorus-based flame retardants, which can act both in the solid phase (promoting the char formation and thus reducing the heat and mass transport between the polymer and the flame zone [14]) and in the gas phase (as radical scavengers [15,16]) offer an effective solution to the fire safety problem of polymeric materials.

In this work, the fire retardancy of carbon fibre reinforced sorbitol polyglycidyl ether (SPE) composites was investigated by applying ammonium polyphosphate (APP), acting in solid phase [14], resorcinol bis(diphenyl phosphate) (RDP), acting primarily in gas phase [17], and their combination, which proved to be synergistic in terms of fire retardancy in SPE polymer matrix according to a paper published recently by the authors [18]. In this work, the fire retardant action of the additive FRs and their synergistic combinations was investigated in carbon fibre–reinforced composites by limiting oxygen index (LOI), UL-94 tests, and mass loss calorimetry. The effect of FRs on the glass transition temperature and storage modulus was evaluated by DMA test. The quasi-static mechanical performance was determined by tensile and three-point bending tests, while the dynamic properties were analysed by Charpy impact test. Interlaminar shear strength (ILSS) of the composites was determined in order to get insight into the quality of the fibre-matrix adhesion.

## 2. Materials and Methods

### 2.1. Materials

A commercially available renewable sugar-based component called sorbitol polyglycidyl ether (SPE), was used as the epoxy (EP) monomer (supplier: Emerald Performance Materials, Moorestown, NJ, USA). Trade name: ERYSIS GE-60, epoxy equivalent weight: 160–190 g/mol, viscosity at 25 °C: 7–9.5 Pas, density at 25 °C: 1.27–1.30 g/cm^3^.

A cycloaliphatic amine MH 3122 (formerly known as T-58) was used as the hardener (supplier: Ipox Chemicals, Budapest, Hungary). Main component: 3,3′-dimethyl-4,4′-diaminodicyclohexylmethane, amine hydrogen equivalent: 60 g/eq, viscosity at 25 °C: 80–120 mPas, density at 25 °C: 0.944 g/cm^3^. Stoichiometric EP component/hardener ratio was applied in all cases, thus the bio-based/synthetic content (mass%) of the SPE matrix was 75/25.

PX35FBUD030 type unidirectional carbon fibre (CF) fabric consisting of Panex 35 50k rovings, with 300 g/m^2^ areal weight (from Zoltek Zrt., Nyergesújfalu, Hungary) was used as the reinforcement.

Resorcinol bis(diphenyl phosphate) (RDP) (supplier: ICL Industrial Products (Beer Sheva, Israel), trade name: Fyrolflex RDP, P-content: 10.7%) and ammonium polyphosphate (APP) (supplier: Nordmann Rassmann (Hamburg, Germany), trade name: NORD-MIN JLS APP, P-content: 31–32%, average particle size of 15 μm) were applied as additive flame retardants.

**Figure 1:**
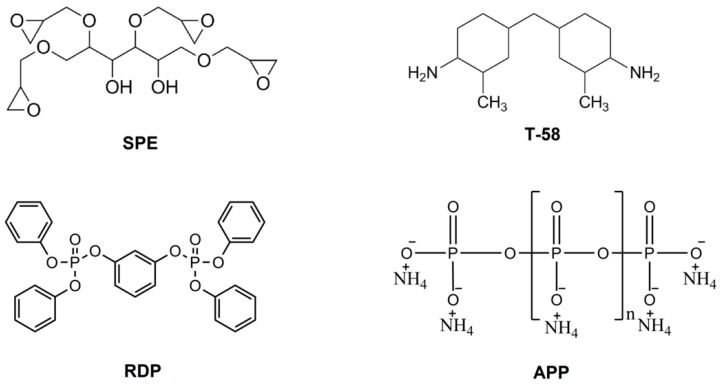
Chemical structures of the applied components.

The chemical structures of the applied EP monomers and FR additives can be seen in Figure 1.

### 2.2. Methods

#### 2.2.1. Composite Sample Preparation

Epoxy resin composites of gradually increasing phosphorus content (1, 2, and 3% related to the matrix) were prepared. First, the flame retardant (FR) component (APP, RDP, or both) was mixed with the SPE epoxy component. Then the curing agent was added and mixed at room temperature in a crystallizing dish in order to obtain a homogenous mixture. The composite laminates were made by hand lamination in a press mould. Each carbon weave layer was separately impregnated. The prepared laminates were put under compression with 180 bar hydraulic pressure (which equals to approx. 25 bar pressure on the laminate) in T30 type platen press (Metal Fluid Engineering s. r. l., Verdello Zingonia, Italy) to achieve high and uniform fibre content in the composites. Two mm thick laminates were made in [0]_5_ layup. The curing procedure, determined on the basis of differential scanning calorimetry (DSC) and gel time tests, consisted of the following isothermal heat steps: 2 h at 80 °C and 2 h at 120 °C. The heat treatment was carried out during pressing. The fibre content of the composites was 60 ± 1 mass%.

#### 2.2.2. Characterization of the Fire Behaviour

The fire behaviour of the reference and flame retarded composites was characterized by limiting oxygen index measurements (LOI, according to ASTM (American Society for Testing and Materials) D2863). The LOI value expresses the lowest volume fraction of oxygen in a mixture of oxygen and nitrogen that supports flaming combustion of a material under specified test conditions. The sample size was 120 mm × 15 mm × 2 mm.

Standard UL-94 flammability tests (according to ASTM D3081 and ASTM D635, respectively) were also carried out in order to classify the samples based on their flammability in horizontal and vertical test setups. The sample size was 120 × 15 × 2 mm. The increasing values of UL-94 ratings are as follows: HB, V-2, V-1, V-0.

Mass loss type cone calorimeter tests were carried out by an instrument made by FTT Inc. (East Grinstead, UK) using the ISO 13927 standard method. Specimens (100 × 100 × 2 mm) were exposed to a constant heat flux of 25 kW/m^2^ and ignited. Heat release values and mass reduction were continuously recorded during burning. 

#### 2.2.3. Dynamic Mechanical Analysis (DMA)

For the investigations of the dynamic mechanical properties and for the determination of the glass transition temperature (T_g_) values DMA tests were carried out in three point bending setup with TA Q800 device of TA Instruments (New Castle, DE, USA). The temperature range was 25–200 °C with 3 °C/min heating rate. The frequency was 1 Hz. The size of the specimens was 55 × 10 × 2 mm (length × width × thickness), and the support span was 50 mm. The amplitude was strain controlled with 0.1% relative strain. From the results glass transition temperature based on the tan delta peaks (T_g_) and storage modulus (E’) values at 25 °C and 75 °C were determined by the software of the device (TA Instruments Universal Analysis 2000 4.7A version).

#### 2.2.4. Tensile Test

Tensile tests were carried out to determine the tensile strength and tensile modulus values by a Zwick Z020 (Ulm, Germany) type computer controlled universal tester, used with a 20 kN capacity load cell and parallel vice grips. Based on EN ISO 527 the specimen size was 140 × 10 × 2 mm (length × width × thickness). The test speed was 5 mm/min, and the initial test length was 100 mm. During the test, force and displacement values were recorded and the tensile parameters were calculated according to the standard.

#### 2.2.5. Bending Test

Bending tests were carried out in three point bending setup to determine the composites flexural strength and flexural modulus values by a Zwick Z020 (Ulm, Germany) type computer controlled universal tester, used with a 20 kN capacity load cell with standard three point bending fixtures. The size of the specimens, based on EN ISO 14125 was 100 × 10 × 2 mm (length × width × thickness). The test speed was 5 mm/min, and the span length was 80 mm. During the test, force and displacement values were recorded and the bending parameters were calculated according to the standard.

#### 2.2.6. Charpy Impact Test

Charpy impact tests were carried out according to EN ISO 179-1 by a normal impact on unnotched specimens of 80 mm length, 10 mm width and 2 mm thick with a Ceast Resil Impactor Junior (Torino, Italy) instrumented pendulum equipped with a 15 J hammer. The force–time curves were registered by a Ceast DAS 8000 data acquisition unit and the Charpy impact energy was calculated.

#### 2.2.7. Interlaminar Shear Test

According to EN ISO 14130 interlaminar shear tests were carried out on 5-5 specimens with 20 × 10 × 2 mm size (length × width × thickness) by a Zwick Z020 (Ulm, Germany) universal tester. The support span was 10 mm and the test speed was 1 mm/min. From the registered force-displacement results apparent interlaminar shear strength (ILSS) was calculated.

## 3. Results and Discussion

### 3.1. LOI and UL-94 Results

The LOI and UL-94 results of the flame retarded composites can be seen in Table 1.

From the two flame retardants, the APP seemed to be more advantageous in terms of LOI: by adding 3% P through APP, LOI of 31 V/V% was reached, while with the same P-content the RDP resulted in LOI of only 27 V/V%. The lower LOI values can be explained by the plasticizing effect induced by high amounts of RDP: due to the relatively low P-content of RDP, higher amount is necessary to reach the same P-content, as in the case of APP. On the other hand, the UL-94 rate ameliorated only in 3% P RDP system among the composites flame retarded only with APP or RDP. Consequently, it can be stated that the gas phase effect (provided by RDP) is indispensable to reduce the ignitability of these composite at ambient oxygen concentrations. As for the combined formulations containing both APP and RDP with overall 3% P-content, the balanced solid and gas-phase flame retardant mechanism (discussed in the case of unreinforced SPE epoxy resin matrix in the previous article of the authors for [18]) lead to LOI of 32 V/V% and V-1 UL-94 rate in the case of RDP 2% P + APP 1% P sample.

In contrast to combined formulations in unreinforced matrix (SPE matrix RDP 1.5% P + APP 1.5% P: 33 V/V% LOI, V-0; SPE matrix RDP 2% P APP 1% P: 34 V/V% LOI [18]), the self-extinguishing, V-0 UL-94 was not reached in carbon fibre composite specimens, most likely due to the so called candlewick effect [19] and intumescent-hindering effect [13] of the introduced reinforcing fibres. Further increase of P-content in order to overcome this issue is not reasonable as at higher ratios the plasticizing effect of RDP becomes significant and the aggregation of solid APP particles is more distinct, which together leads to lower crosslinking density and impairs the flame retardant and mechanical performance.

### 3.2. Mass Loss Calorimetry Results

**Figure 2:**
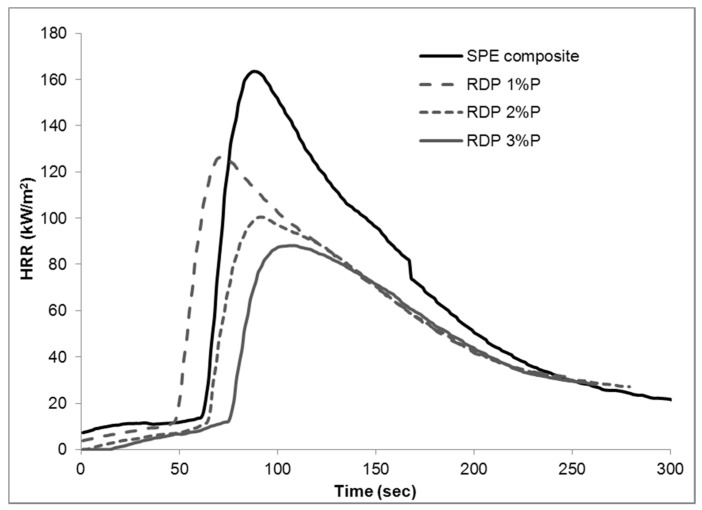
Heat release rate of reference and flame retarded SPE composites with resorcinol bis(diphenyl phosphate) (RDP).

**Figure 3:**
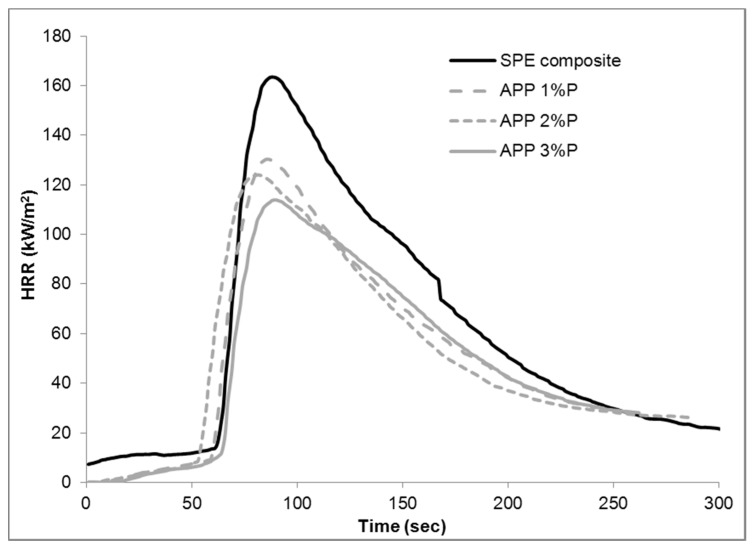
Heat release rate of reference and flame retarded SPE composites with ammonium polyphosphate (APP).

**Figure 4:**
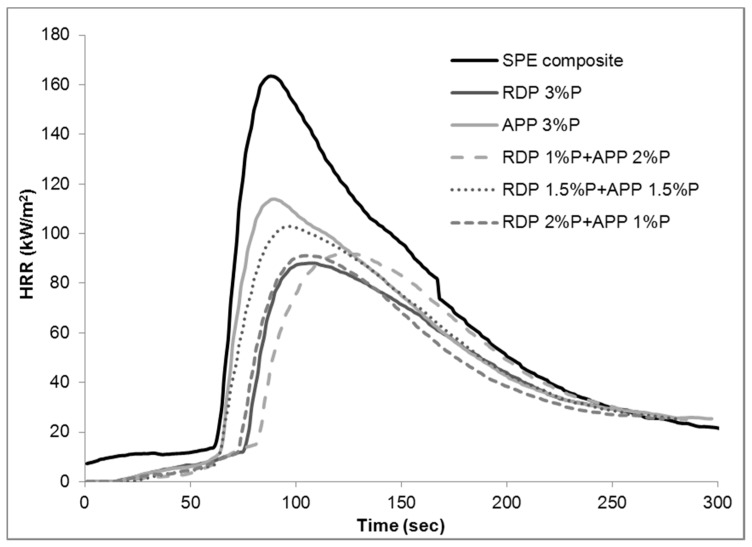
Heat release rate of reference and flame retarded SPE composites with 3% phosphorus.

The heat release rate curves obtained from mass loss calorimetry tests can be seen in Figure 2 (samples flame retarded with RDP), Figure 3 (samples flame retarded with APP), and in Figure 4 (samples containing 3% P including the mixed formulations). Numerical data obtained from mass loss calorimetry results are summarized in Table 2, where the best performances are highlighted with bold letters.

In the case of samples flame retarded by RDP, the pHRR decreased gradually from 163 to 88 kW/m^2^ by increasing the amount of RDP. At 1% P-content, the sample ignited earlier and reached the time of pHRR before the SPE reference, but at higher concentrations the flame retardant effect prevailed over reduced thermal stability at early stage of degradation [18]: both the TTI and time of pHRR increased (at 3% P-content by 16 s and 19 s, respectively, compared to the SPE reference). In cases of total heat release (THR), fire growth rate (FIGRA), maximum of average of heat emission (MARHE), and effective heat of combustion (EHC), the determined values decreased with increasing P-content, and the highest reduction was reached when the P-content was doubled from 1 to 2% P (e.g., FIGRA decreased from 1.8 kW/m^2^s to 1.1 kW/m^2^s when the P-content increased from 1 to 2%, while at 3% P only a further reduction of 0.3 kW/m^2^s to 0.8 kW/m^2^s was observed). Composites having larger amount of P incorporated showed higher residual mass after combustion.

In APP-containing samples, the best results were also achieved at 3% P, but the additive induced improvements were less significant than in the case of RDP. Regarding the APP 2% P sample, both the TTI and pHRR appears earlier than in the case of the 1 and 3% P-containing samples, while no significant reduction compared to APP 1% P was observed in the case of FIGRA, THR, and EHC. Despite the increasing P-content of the different composites, no difference was observed in the values of the residual masses either. According to the literature APP has pure solid phase mechanism, while RDP has main gas phase effect with some minor solid phase one; consequently by adding a flame retardant acting in the gas phase, major improvements were expected in the case of APP.

All mixed formulations showed better performance than 3% P APP: by changing the origin of 1% P from APP to RDP, the TTI increased by 12 s, pHRR and EHC decreased by approx. 20% and FIGRA by approx. 40%. Comparing the combined formulations to the RDP 3% sample, some slight improvements could be achieved e.g., in RDP 1% P + APP 2% P the TTI increased by 7 s and the time of pHRR by 15 s, and the EHC was reduced, while in RDP 2% P + APP 1% P THR, EHC and MARHE were slightly reduced. These observations also reveal that it is advantageous, if the applied flame retardant or flame retardant combination performs action in both gas and solid phase [18,20].

Considering the overall fire retardancy performance of the composites, the combined RDP 2% P + APP 1% P formulations can be considered as an optimum. It has the highest LOI (32 V/V%), reaches V-1 rating (on 2 mm thick composites) in UL-94 test, the pHRR is decreased by 45% compared to the reference SPE composite, it has the lowest THR and MARHE values, and it has the highest residual mass among the tested compositions. Further improvement could be achieved probably by forming coated fibre-reinforced composite according to the results published by the authors recently [13].

### 3.3. Glass Transition Temperature

**Figure 5:**
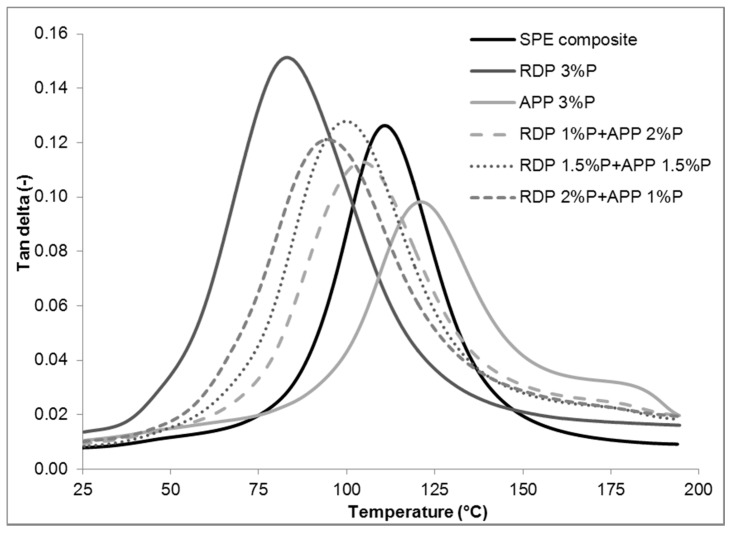
Tan delta curves of reference and 3% P-containing SPE composites.

In general, the application of flame retardants influences the glass transition temperature (T_g_) of polymer composites significantly, and subsequently their applicability as well. The tan delta curves of the reference and 3% P-containing composites in the function of temperature are displayed in Figure 5, while the T_g_ of the flame retarded SPE composites determined from tan delta peaks can be seen in Table 3.

Similarly to the results in SPE matrices published previously by the authors [18], the low P-containing RDP has a significant plasticizing effect in SPE composites. By increasing the amount of RDP, the T_g_ gradually decreased. At 3% P from RDP the decrease in T_g_ is 28 °C. In contrast, the T_g_ of the composites increased when APP was introduced. This can be explained on the one hand by the higher P-content of APP (meaning that less amount is needed to reach the same P-content than in the case of RDP), and on the other hand well-dispersed solid APP particles can block the segmental movements in the cross-linked epoxy resins [21]. In mixed FR formulations, the T_g_ decrease (caused by RDP) was partially compensated by APP.

The measured values of tan delta (ratio of loss modulus to storage modulus) give information about the damping characteristics of the materials. As it can be seen in Table 3, with increasing amount of RDP, the tan delta values increase, meaning that the damping capability of the composite increases due to easier segmental movements (by 20% when the RDP is applied in a concentration corresponding to 3% P content). Contrarily, when APP is applied as flame retardant, the tan delta decreases as the segmental movements and thus the energy dissipation ability of the polymer chains are blocked by the solid FR particles. In the case of the mixed formulations, the different effects of the liquid RDP and the solid APP compensate each other leading to similar tan delta values as the reference SPE composite.

### 3.4. Storage Modulus of the Composites

**Figure 6:**
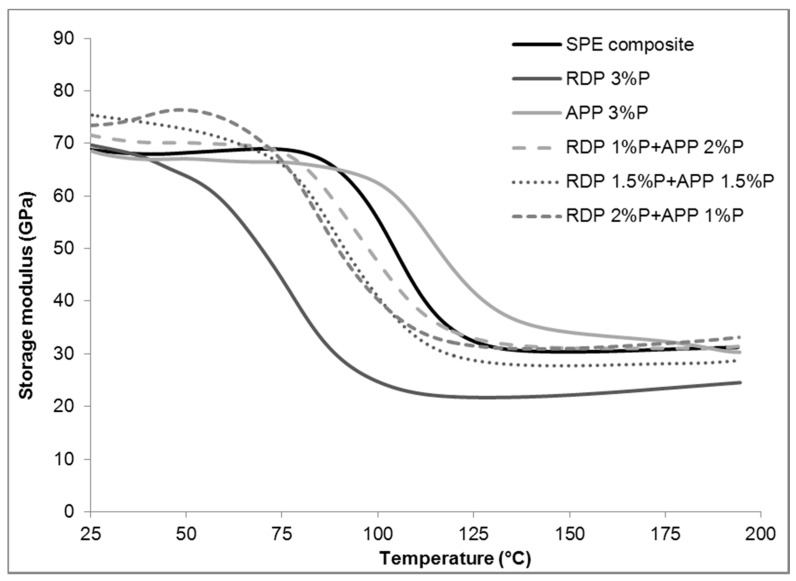
Storage modulus curves of reference and flame retarded SPE composites.

Storage modulus curves of reference and 3% P-containing flame retarded SPE composites are displayed in Figure 6, while numerical results of dynamical mechanical analysis (storage modulus at 25 °C and 75 °C) of composites are shown in Table 4.

As can be seen from the results, by increasing the amount of RDP in SPE composites, the storage modulus decreases in the whole temperature region of the measurement (25–200 °C), which can be explained by significant plasticizing effect of the liquid RDP. Due to the relatively low P-content of RDP, for appropriate flame retardancy high amount is needed, resulting in gradually decreasing moduli with increasing FR content. Comparing the modulus values measured at room temperature (25 °C) and at elevated temperature below T_g_ (75 °C), it can be stated that the reference and the APP containing composites show only negligible decrease, while samples containing RDP (alone or in combination with APP) have significantly reduced moduli at higher temperatures. In the composites containing both APP and RDP, up to approximately 70 °C the storage modulus is higher than in the case of SPE reference, while above this temperature the plasticizing effect of RDP prevails over the reinforcing effect of APP leading to decreasing storage modulus.

### 3.5. Mechanical Properties of the Composites

The tensile, flexural, interlaminar shear, and Charpy impact properties of the reference and flame retarded SPE composites are summarized in Table 5.

As for the tensile strength values, it can be concluded that in the case of composites containing only one FR, by increasing the amount of FR, the tensile strength showed an increasing tendency, but all values were slightly below the reference SPE composite. The tensile strength of the mixed FR formulations overperformed this reference, especially in the case of RDP 1% P + APP 2% P composite, and showed an increasing tendency with the increase of APP ratio. Concerning the flexural strength results, the composites containing only APP or RDP had somewhat lower values than the SPE reference. The mixed FR samples had higher flexural strength than the composites with the same amount of P originating from one FR, and fell in the range of standard deviation of the SPE reference composite. The samples containing only RDP showed somewhat higher ILSS values than the SPE composite reference, while the samples containing APP and the mixed formulations were in the same range as the reference, no clear tendencies could be observed. Concerning the dynamic properties, in the case of samples containing only RDP or APP, the Charpy impact energy increased by increasing the amount of the FR. All samples with 3% P had higher values than the reference SPE composite, highest value was achieved in the case of APP 3% P, followed by RDP 1% P + APP 2% P sample. Furthermore, in mixed FR samples, the Charpy impact energy increased by increasing the ratio of APP.

## 4. Conclusions

In this work, the fire retardancy of carbon fibre reinforced sorbitol polyglycidyl ether (SPE) bioepoxy composites was investigated by applying liquid resorcinol bis(diphenyl phosphate) (RDP), solid ammonium polyphosphate (APP), and their combinations.

According to the literature, APP acts only in solid phase, while RDP mainly has a gas phase effect (with some minor solid phase effect). For this reason, major improvements were expected in the case of APP by adding a complementary gas phase FR effect by introducing RDP. Concerning the fire retardancy results, at ambient oxygen concentrations the gas phase effect of RDP was essential to reduce the ignitability of the composite: LOI of 32 V/V% and V-1 UL-94 rate in the case of RDP 2% P + APP 1% P mixed formulation was reached. As for the heat release rate results, the combined RDP 2% P + APP 1% P composite can be considered as an optimum.

The low P-containing RDP has a significant plasticizing effect in SPE composites resulting in decreased T_g_ and storage modulus. On the other hand, as well-dispersed solid APP particles can block the segmental movements in the cross-linked epoxy resins, APP increased the T_g_ of the composites and partially compensated the T_g_ decrease caused by RDP in mixed FR formulations and resulted in higher storage modulus values up to 75 °C in all combined formulations than that of SPE reference.

The application of FRs usually decreases the mechanical properties of polymer composites. When applying APP or RDP alone, the tensile and flexural strength of the composites decreased. However, mixed FR formulations led to higher tensile strength than that of the reference SPE composite, and their flexural strength also remained in the region of the reference. Concerning the dynamic properties, all composites with 3% P content had higher values than the SPE reference. In composites containing both RDP and APP, the Charpy impact energy increased by increasing the ratio of APP. Accordingly, the combination of the liquid RDP and solid APP proved to be an effective concept to improve the FR performance and maintain/increase the mechanical properties of carbon fibre reinforced bioepoxy composites at the same time.

## Figures and Tables

**Table 1:** Limiting oxygen index (LOI) and UL-94 results of the flame retarded sorbitol polyglycidyl ether (SPE) composites.

Flame Retardant Composition	LOI [V/V%]	UL-94
SPE composite reference	24	HB (1st ignition)
RDP 1% P	26	HB (1st ignition)
RDP 2% P	26	HB (1st ignition)
RDP 3% P	27	V-1
APP 1% P	25	HB (1st ignition)
APP 2% P	28	HB (1st ignition)
APP 3% P	31	HB (2nd ignition)
RDP 1% P + APP 2% P	30	HB (2nd ignition)
RDP 1.5% P + APP 1.5% P	31	HB (2nd ignition)
RDP 2% P + APP 1% P	32	V-1

**Table 2:** Mass loss type cone calorimetry results of reference and flame retarded SPE composites.

Flame Retardant Composition	TTI [s]	pHRR [kW/m^2^]	Time of pHRR [s]	FIGRA [kW/m^2^s]	THR [MJ/m^2^]	EHC [MJ/kg]	MARHE [kW/m^2^]	Residue [mass%]
SPE composite reference	61	163	88	1.9	16.9	16.4	77.4	60
RDP 1% P	57	127	72	1.8	14.4	15.7	66.3	63
RDP 2% P	66	100	91	1.1	11.9	13.2	51.4	65
RDP 3% P	77	**88**	107	**0.8**	10.6	12.3	44.3	67
APP 1% P	61	130	86	1.5	13.4	14.4	60.3	65
APP 2% P	58	124	81	1.5	13.3	14.1	61.7	64
APP 3% P	72	114	90	1.3	12.7	13.5	55.7	65
RDP 1% P + APP 2% P	**84**	92	**122**	**0.8**	10.9	**11.0**	45.2	63
RDP 1.5% P + APP 1.5% P	64	103	97	1.1	12.1	13.3	52.3	65
RDP 2% P + APP 1% P	72	91	105	0.9	**10.3**	12.2	**44.1**	**68**

TTI: time to ignition, pHRR: peak of heat release rate, FIGRA: fire growth rate, THR: total heat release, EHC: average effective heat of combustion, MARHE: maximum of average rate of heat emission, average standard deviation of the measured mass loss calorimeter values: TTI: ±3, pHRR: ±30, time of pHRR: ±5, residue: ±2.

**Table 3:** Effect of the additive flame retardants on the glass transition temperature (T_g_) of SPE composites.

Flame Retardant Composition	tan delta [-]	T_g_ [°C]
SPE composite reference	0.1263	111
RDP 1% P	0.1385	102
RDP 2% P	0.1417	94
RDP 3% P	0.1513	83
APP 1% P	0.0997	115
APP 2% P	0.0983	120
APP 3% P	0.0982	121
RDP 1% P + APP 2% P	0.1128	104
RDP 1.5% P + APP 1.5% P	0.1279	100
RDP 2% P + APP 1% P	0.1211	95

**Table 4:** Storage moduli of reference and flame retarded SPE composites.

Flame Retardant Composition	Storage Modulus at 25 °C [GPa]	Storage Modulus at 75 °C [GPa]
SPE composite reference	68.79	68.86
RDP 1% P	67.33	61.76
RDP 2% P	65.04	58.93
RDP 3% P	69.60	44.23
APP 1% P	80.33	76.85
APP 2% P	62.18	61.31
APP 3% P	68.40	66.43
RDP 1% P + APP 2% P	71.54	68.33
RDP 1.5% P + APP 1.5% P	75.34	65.96
RDP 2% P + APP 1% P	73.48	66.70

**Table 5:** Tensile, flexural, interlaminar shear, and Charpy impact properties of reference and flame retarded SPE composites.

Flame Retardant Composition	Tensile Strength [MPa]	Flexural Strength [MPa]	Interlaminar Shear Strength [MPa]	Charpy Impact Energy [kJ/m^2^]
SPE reference	916.2 ± 18.7	996.9 ± 64.3	41.1 ± 1.9	85.5 ± 6.6
RDP 1% P	818.8 ± 56.5	867.7 ± 62.2	44.3 ± 1.3	88.7 ± 6.9
RDP 2% P	826.4 ± 49.8	900.4 ± 64.9	47.5 ± 0.7	90.5 ± 19.3
RDP 3% P	851.2 ± 29.7	919.5 ± 138.4	41.3 ± 1.0	93.0 ± 6.9
APP 1% P	795.6 ± 123.0	895.0 ± 41.3	42.7 ± 1.5	75.1 ± 8.9
APP 2% P	842.1 ± 49.9	948.4 ± 43.4	41.0 ± 1.4	79.8 ± 9.4
APP 3% P	914.3 ± 76.6	927.4 ± 74.4	42.7 ± 1.2	102.2 ± 33.6
RDP 1% P + APP 2% P	1025.0 ± 23.4	1004.3 ± 95.2	41.0 ± 2.8	99.0 ± 19.9
RDP 1.5% P + APP 1.5% P	951.1 ± 37.3	956.0 ± 98.5	41.0 ± 1.1	95.0 ± 17.6
RDP 2% P + APP 1% P	948.1 ± 8.3	976.0 ± 98.5	38.3 ± 1.2	87.0 ± 1.8
